# The Impact of Serum Glucose on the Predictive Value of Serum Lactate for Hospital Mortality in Critically Ill Surgical Patients

**DOI:** 10.1155/2019/1578502

**Published:** 2019-11-26

**Authors:** Xue Chen, Jianbin Bi, Jia Zhang, Zhaoqing Du, Yifan Ren, Shasha Wei, Fenggang Ren, Zheng Wu, Yi Lv, Rongqian Wu

**Affiliations:** ^1^National Local Joint Engineering Research Center for Precision Surgery & Regenerative Medicine, Shaanxi Provincial Center for Regenerative Medicine and Surgical Engineering, First Affiliated Hospital of Xi'an Jiaotong University, Xi'an, Shaanxi Province, China; ^2^Department of Hepatobiliary Surgery, First Affiliated Hospital of Xi'an Jiaotong University, Xi'an, Shaanxi Province, China

## Abstract

**Background:**

Lactate has been widely used as a risk indicator of outcomes in critically ill patients due to its ready measurement and good predictive ability. However, the interconnections between lactate metabolism and glucose metabolism have not been sufficiently explored, yet. In this study, we aimed to investigate whether glucose levels could influence the predictive ability of lactate and design a more comprehensive strategy to assess the in-hospital mortality of critically ill patients.

**Methods:**

We analyzed the clinical data of 293 critically ill patients. The primary outcome was in-hospital mortality. The logistic regression analysis and the area under the receiver operating characteristic curve (AUROC) were applied to evaluate the predictive ability of lactate in association with glucose.

**Results:**

The lactate level showed significant association with in-hospital mortality, and its predictive ability was also comparable to other prognostic scores such as the SOFA score and APACHE II score. We further divided 293 patients into three groups based on glucose levels: low-glucose group (<7 mmol/L), medium-glucose group (7-9 mmol/L), and high-glucose group (>9 mmol/L). The lactate level was associated with in-hospital mortality in the low- and high- glucose groups, but not in the medium-glucose group, whereas the SOFA score and APACHE II score were associated with in-hospital mortality in all three glucose groups. The AUROC of lactate in the medium-glucose group was also the lowest among the three glucose groups, indicating a decrease in its predictive ability.

**Conclusions:**

Our findings demonstrated that the predictive ability of lactate to assess in-hospital mortality could be influenced by glucose levels. In the medium glucose level (i.e., 7-9 mmol/L), lactate was inadequate to predict in-hospital mortality and the SOFA score; the APACHE II score should be utilized as a complementation in order to obtain a more accurate prediction.

## 1. Background

An elevated lactate level is a well-known predictor of organ dysfunction and mortality in critically ill patients [1–10]. The pathways for glucose and lactate metabolism are deeply interconnected. In the Cori cycle, glucose can be converted to lactate though glycolysis and lactate can generate glucose through gluconeogenesis, indicating that glucose can greatly influence lactate metabolism and vice versa. In this regard, blood glucose levels may be a confounder in the association between high lactate levels and increased mortality in ICU patients. Some studies have demonstrated the associations of glucose level and lactate level with the risk of death [1, 3, 5, 7–17]. A recent study has shown that a low glucose level combined with a high lactate level was associated with the highest mortality of ICU patients [11]. However, whether glucose levels influence the predictive value of lactate for hospital mortality in critically ill patients remained largely unknown. Herein, we conducted a study to investigate the predictive ability of lactate at various glucose levels. Specifically, we hypothesized that glucose levels could modify the association between high lactate levels and increased mortality in critically ill patients. We aimed to get a more comprehensive appreciation and a more accurate prediction strategy of using lactate as a prognostic biomarker to assess the outcomes of ICU patients.

## 2. Methods

### 2.1. Patients and Data Sources

We conducted a retrospective study of 293 patients older than 18 years of age admitted to the intensive care unit (ICU) of The First Affiliated Hospital of Xi'an Jiaotong University from June 2013 to Dec 2016. The study was approved by the medical ethics committee of Xi'an Jiaotong University. The patient's informed written consent was waived due to the retrospective nature of this study. Anonymized patient information was obtained from the hospital's electronic patient database. The following data were recorded from the electronic medical record: demographics, comorbidities, initial and worst vital signs, and laboratory measurements. The APACHE II score, SOFA score, and qSOFA score were calculated according to References [18–20]. Renal dysfunction was defined by the presence of acute kidney injury (AKI) according to the Kidney Disease Improving Global Outcomes (KDIGO) criteria [21].

### 2.2. Statistical Analysis

Continuous data was tested for normality by the Kolmogorov-Smirnov test. Normal distribution variables are reported as the means ± standard deviations (SD) and compared by Student's *t*-test. Abnormal distribution variables are reported as the medians (interquartile range (IQR)) and compared by the Mann–Whitney rank-sum test. Categorical variables are reported as the numbers and percentages and compared by the chi-squared analysis or Fisher's exact test. *p* < 0.05 was considered to be statistically significant. The logistic regression analysis and the area under the receiver operating characteristic curve (AUROC) were applied to evaluate the predictive ability of lactate. All statistics analyses were performed using IBM SPSS version 23.0 for Windows (IBM, Chicago, Ill., United States).

## 3. Results

### 3.1. Patient Characteristics

We analyzed the data of 293 patients admitted to the ICU between June 2013 and Dec 2016. Of these patients, 59 (20.1%) died during hospital stay. Patient characteristics of hospital survivors and nonsurvivors are shown in Table 1. There were no statistically significant differences in age, gender, and reason for ICU admission between survivors and nonsurvivors. In terms of comorbidities, the deceased patients were more likely to have diabetes mellitus and multiple organ failure (MOF) than survivors. In addition, the lactate level, SOFA score, and APACHE II score at ICU admission were significantly higher in nonsurvivors than in survivors. However, there was no significant difference in blood glucose levels between the hospital survivors and nonsurvivors.

### 3.2. Associations of Patient's Clinical Characteristics and Predictive Scores with In-Hospital Mortality

We firstly conducted univariable logistic regression analysis to investigate the associations of patients' clinical characteristics and predictive scores (including lactate level, glucose level, SOFA score, APACHE II score, and qSOFA score) with in-hospital mortality. As shown in Table 2, patients' age, gender, and reason for ICU admission were unassociated with in-hospital mortality. Among 15 various comorbidities, patients with diabetes mellitus and patients with MOF correlated significantly with higher in-hospital mortality. Among the five predictive scores, lactate level, SOFA score, APACHE II score, and qSOFA score were strongly associated with in-hospital mortality whereas the glucose level was not associated with in-hospital mortality. We further conducted a multivariate logistic regression analysis using the parameters shown to have statistical significance by univariate analysis. As a result, only diabetes mellitus, APACHE II, and lactate level were correlated significantly with higher in-hospital mortality.

**Figure 1:**
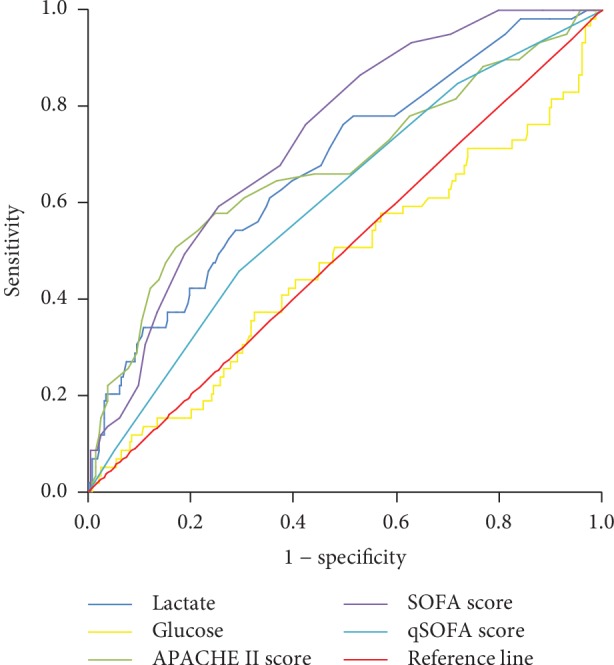
ROC of lactate, glucose, APACHE II score, SOFA score, and qSOFA score. The ROC of the five indexes were plotted, and their AUROC were compared. AUROC of lactate, glucose, APACHE II score, SOFA score, and qSOFA score were 0.678, 0.472, 0.675, 0.736, and 0.605, respectively.

The receiver operating characteristic (ROC) curve was also applied in our study to assess the mortality prediction performance of the five predictive scores. As seen in Figure 1, the AUROC of lactate level, glucose level, APACHE II score, SOFA score, and qSOFA score were 0.678, 0.472, 0.675, 0.736, and 0.605, respectively. The AUROC of lactate was the second largest and comparable to that of the SOFA score. The calculated cutoff for lactate to predict in-hospital mortality was 1.45 mmol/L. The AUROC of glucose, on the other hand, was much lower than that of the other four scores.

### 3.3. Glucose Levels Influence the Predictive Ability of Lactate

**Figure 2:**
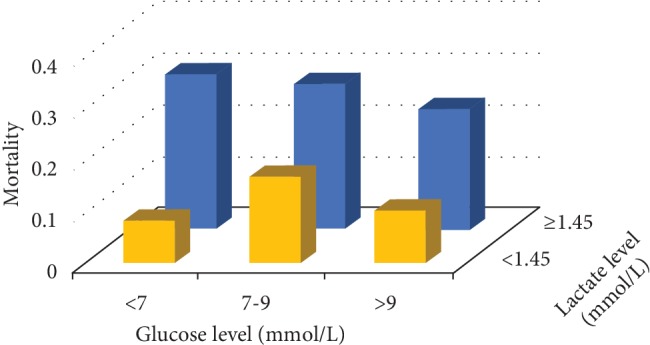
Combined effect of lactate and glucose on in-hospital mortality. The highest mortality was observed in the group with the lowest glucose level and the highest lactate level.

**Figure 3:**
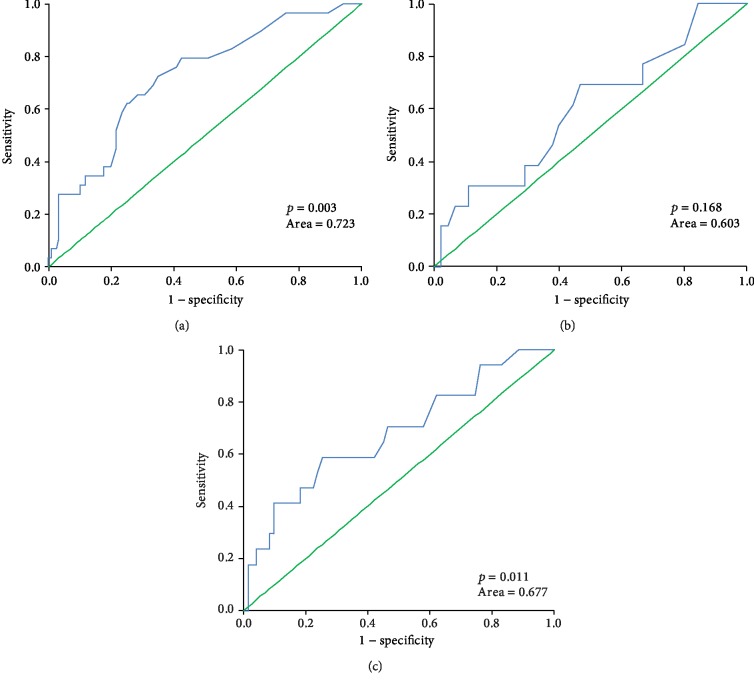
ROC of lactate in the three glucose groups: (a) the ROC of lactate in the low-glucose group (glucose < 7 mmol/L) with an AUROC of 0.723; (b) the ROC of lactate in the medium-glucose group (7 mmol/L ≤ glucose ≤ 9 mmol/L) with an AUROC of 0.603; (c) the ROC of lactate in the high-glucose group (glucose > 9 mmol/L) with an AUROC of 0.677.

It has been reported that the glucose showed a U-shaped characteristic in a number of illnesses [12, 19]. The “safe range” of glucose has been defined between 7.0 mmol/L and 9.0 mmol/L (the conversion factor of glucose is 1 mmol/L = 18 mg/dL) in critically ill patients [11, 12]. Following these criteria, we divided the 293 patients into three groups based on the glucose levels: low-glucose group (glucose < 7 mmol/L), medium-glucose group (glucose: 7-9 mmol/L), and high-glucose group (glucose > 9 mmol/L). Each group was further divided into two subgroups based on lactate cutoff point as shown in [Supplementary-material supplementary-material-1] (i.e., 1.45 mmol/L; the conversion factor of lactate is 1 mmol/L = 9 mg/dL). The association of lactate levels with in-hospital mortality and the predictive ability of lactate were investigated in this scenario. As seen in Figure 2, the highest mortality was observed in the group with the lowest glucose level and the highest lactate level. The lactate level was associated with in-hospital mortality in the low- and high-glucose groups (*p* < 0.001), but not in the medium-glucose group (Table 3). The AUROC of lactate in the three glucose groups were 0.723, 0.603, and 0.677, respectively. The AUROC of lactate in the medium-glucose group was also the lowest among the three (Figure 3).

### 3.4. APACHE II Score and SOFA Score Could Be Utilized as an Alternative Predictor of Hospital Mortality in the Medium-Glucose Group

**Figure 4:**
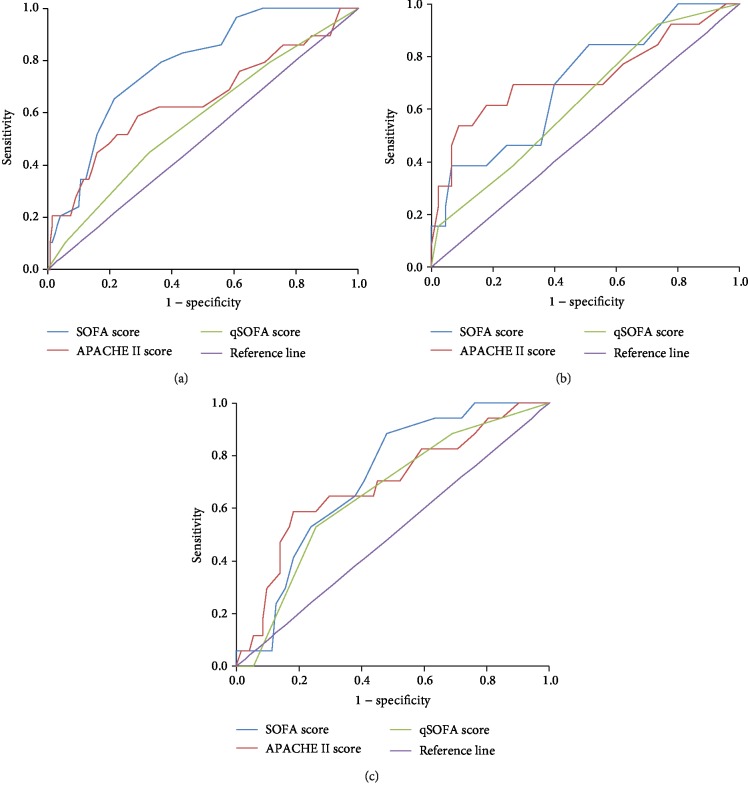
ROC of the APACHE II score, SOFA score, and qSOFA in the three glucose groups: (a) in the low-glucose group (glucose < 7 mmol/L), the AUROC of the APACHE II score, SOFA score, and qSOFA score were 0.650, 0.779, and 0.574, respectively; (b) in the medium-glucose group (7 mmol/L ≤ glucose ≤ 9 mmol/L), the AUROC of the APACHE II score, SOFA score, and qSOFA score were 0.723, 0.700, and 0.629, respectively; (c) in the high-glucose group (glucose > 9 mmol/L), the AUROC of the APACHE II score, SOFA score, and qSOFA score were 0.690, 0.712, and 0.652, respectively.

In addition to lactate, the association between patients' clinical characteristics and other predictive scores (including APACHE II score, SOFA score, and qSOFA score) with in-hospital mortality in the three glucose groups was also investigated. As seen in Table 3, only the SOFA score and APACHE II score were clearly associated with higher in-hospital mortality in all three glucose groups whereas the association of other indexes with mortality was influenced by glucose levels. The ROC and AUROC of the SOFA score, APACHE II score, and qSOFA score were also plotted and compared with that of lactate as show in Figure 4. In the medium-glucose group where the lactate level was unassociated with in-hospital mortality, the APACHE II score presented the largest AUROC, followed by the SOFA score, qSOFA, score and lactate.

## 4. Discussion

In this study, we demonstrated that glucose levels could influence the association between lactate and in-hospital mortality in critically ill patients. Specifically, in the low- (less than 7 mmol/L) and high- (more than 9 mmol/L) glucose groups, lactate was strongly associated with in-hospital mortality and could provide a good performance in predicting in-hospital mortality. In the medium-glucose group (between 7 mmol/L and 9 mmol/L), however, lactate was unassociated with in-hospital mortality and its predictive ability reduced significantly. Moreover, we also demonstrated that the APACHE II score and SOFA score were correlated with higher in-hospital mortality in all three glucose groups. Therefore, in patients with glucose levels between 7 and 9 mmol/L where the lactate level was inadequate to predict in-hospital mortality, the SOFA score and APACHE II score could be utilized as alternative indexes in order to achieve a more accurate prediction.

Glucose and lactate intervene in a very complex way in a series of glycometabolic pathways and play important roles in glycometabolic related diseases. Plenty of observational studies have demonstrated that the glucose level during ICU admission was related to mortality by a U-shaped curve [11, 12]. Both hyperglycemia and hypoglycemia had an increased risk of death and were associated with an increase in the ICU length of stay [13–16]. The “safe range” of glucose has been defined between 7.0 and 9.0 mmol/L which is within the optimal blood glucose concentration (BGC) target range (NICE-SUGAR protocol) [12, 13]. The mean glucose values between 7.0 and 9.0 mmol/L during ICU stay were associated with the lowest OR for mortality at the ICU, while the mean values below 7.0 and higher than 9.0 mmol/L conferred significantly higher ORs [12].

Lactate has also been widely studied as a biomarker of the outcomes of a series of disease related to tissue hypoxia, sepsis, and organ failure [1, 3–5, 7–10, 17, 22]. Bakker et al. reported that patients with lower organ failure scores had a lower initial blood lactate level and a shorter duration of lactic acidosis, while patients who died during the first 24 hours had a higher initial blood lactate level and a longer duration of lactic acidosis [9]. Amorini et al. utilized serum lactate as a monitoring of “virtual hypoxia” in multiple sclerosis patients and a secondary outcome for treatment trials aimed at improving mitochondrial function in patients with multiple sclerosis [10]. Krinsley et al. demonstrated that the hypoglycemia could be the result of impaired renal and liver function which affects outcomes [4]. Filho et al. reported the utilization of cerebrospinal fluid (CSF) lactate as a potential biomarker to distinguish between children with bacterial and aseptic meningitis [5]. Mokline et al. reported that the plasma lactate could be used as a powerful predictor biomarker of sepsis and mortality in burn patients. An initial lactate value of 4 mmol/L provided the best sensitivity (88%) and specificity (79%), and the cutoff value for mortality prediction was 4.46 mmol/L with a good sensitivity (86%) and specificity (92%) [3]. Mikkelsen et al. demonstrated that the initial serum lactate was associated with mortality in patients admitted to the ED with severe sepsis [8]. Shapiro et al. demonstrated that lactate level greater than or equal to 4.0 mmol/L had good sensitivity and specificity to predict the mortality within 3 days [7].

Many effects have been done in investigating the relations of lactate and glucose with the outcomes of ICU patients; however, the combined effect of glucose and lactate has rarely been well studied. A recent study conducted by Jorge et al. investigated the relation between the combined glucose/lactate and mortality as well as organ failure. They found that the combination of the highest lactate quintile with the lowest glucose quintile was associated with the highest rates of renal dysfunction, liver dysfunction, and mortality [11]. In addition to glucose, interactions between lactate and other parameters have also been studied. Ospina-Tascón et al. demonstrated that the combined Cv-aCO_2_/Da-vO_2_ ratio with lactate levels could better identify patients at a high risk of adverse outcomes during early stages of resuscitation of septic shock [23]. Ho and Lan reported that combining the qSOFA score with plasma lactate had a predictive ability comparable to the standard SOFA score [24].

Our study substantially extends these previous investigations and presented a comprehensive view of how the overall glycometabolic metabolism affects the outcomes of ICU patients. We demonstrated that prediction of in-hospital mortality of ICU patient is glycometabolism related rather than only lactate related. Neither glucose nor lactate could be used as a predictor of the in-hospital mortality independently. In the low- and high-glucose groups, lactate was associated significantly with in-hospital mortality, whereas in the medium-glucose group, lactate was inadequate to provide a good prediction of in-hospital mortality and other well-accepted indexes such as the APACHE II score and SOFA score should be utilized to obtain a more reasonable and accurate assessment of in-hospital mortality.

Nevertheless, some limitations could still be seen in our study. Due to the small number of clinical samples, selection bias might be inevitable and influence the statistical results. It is interesting to note that in the low- (less than 7 mmol/L) glucose group, diabetes mellitus has a near significant *p* value (0.053), indicating that at low glucose levels, diabetes mellitus may be also associated with the in-hospital mortality. This finding is consistent with previous demonstration that the low glucose is associated with the higher risk of death [25–27]. Therefore, we envisioned that with larger populations, a more reasonable and comprehensive demonstration could be generated. Moreover, as it is retrospective and one center study, prospective and multicenter researches would be required in the future to further validate our conclusions.

## 5. Conclusion

In conclusion, our results showed that the predictive ability of lactate to assess in-hospital mortality also depended on glucose levels. In low and high glucose levels, lactate level would provide a good prediction of in-hospital mortality while in the medium glucose level, the APACHE II score or SOFA score could provide a more accurate and more comprehensive prediction of in-hospital mortality.

## Figures and Tables

**Table 1:** Patient characteristics, reason for ICU admission and comorbidities.

Variable	All patients (*n* = 293)	Hospital survivors (*n* = 234)	Hospital nonsurvivors (*n* = 59)	*p* value
Age	61.44	60.87	63.71	0.306
Male	62.8% (184)	62.7% (147)	62.8% (37)	0.988
Reason for ICU admission				
Cardiovascular disease	17.4% (51)	15.4% (36)	25.4% (15)	0.069
Gastrointestinal disease	27.6% (81)	25.6% (60)	35.6% (21)	0.127
Neurological disease	14.7% (43)	13.7% (32)	18.6% (11)	0.335
Respiratory disease	26.3% (77)	25.6% (60)	28.8% (17)	0.621
Surgery	34.5% (101)	34.6% (81)	33.9% (20)	0.918
Sepsis	13.7% (40)	14.1% (33)	11.9% (7)	0.655
Trauma	10.6% (31)	11.5% (27)	6.8% (4)	0.288
Other	6.5% (19)	5.1% (12)	11.9% (7)	0.060
Comorbidities				
ACS	10.6% (31)	9.4% (22)	15.3% (9)	0.192
AKI	16.7% (49)	17.1% (40)	15.3% (9)	0.735
ALI	10.6% (31)	10.3% (24)	11.9% (7)	0.720
ARDS	13.3% (39)	12.4% (29)	16.9% (10)	0.357
Cirrhosis	8.9% (26)	9.4% (22)	6.8% (4)	0.572
Diabetes mellitus	14.0% (41)	11.5% (27)	23.7% (14)	**0.016**
DIC	1.4% (4)	1.3% (3)	1.7% (1)	0.807
Drinking	22.5% (69)	25.6% (60)	10.2% (9)	0.093
Hypertension	28.3% (83)	29.1% (68)	25.4% (15)	0.580
Malignant disease	22.1% (65)	21.8% (51)	23.7% (14)	0.749
MOF	16.7% (49)	14.1% (33)	27.1% (16)	**0.017**
Smoking	32.4% (95)	34.2% (80)	25.4% (15)	0.199
Stroke	13.0% (38)	12.8% (30)	13.6% (8)	0.880
APACHE II at ICU admission	16.55	15.46	20.90	**<0.001**
Glucose level at ICU admission	8.02	8.06	7.85	0.505
Lactate level at ICU admission	3.82	2.81	5.64	**<0.001**
qSOFA score at ICU admission	1.29	1.06	1.39	**0.008**
SOFA score at ICU admission	8.02	7.33	10.76	**<0.001**
Length of ICU stay	14.62	13.91	17.42	0.355

*p* values generated by either Mann–Whitney or *χ*^2^ test. ACS: acute coronary syndrome; AKI: acute kidney injury; ALI: acute lung injury; ARDS: acute respiratory distress syndrome; DIC: disseminated intravascular coagulation; IHD: ischemic heart disease; APACHE II: Acute Physiology and Chronic Health Evaluation II; SOFA: Sequential Organ Failure Assessment; qSOFA: Quick Sequential Organ Failure Assessment.

**Table 2:** Univariate and multivariate analyses of in-hospital mortality.

Variable	Univariable regression		Multivariable regression	
	OR (95%-CI)	*p* value	OR (95%-CI)	*p* value
Age	1.010 (0.993-1.027)	0.266		
Gender	0.995 (0.551-1.797)	0.988		
Reason for ICU admission				
Cardiovascular disease	1.875 (0.945-3.720)	0.072		
Gastrointestinal disease	0.624 (0.240-1.147)	0.129		
Neurological disease	1.447 (0.681-3.074)	0.337		
Respiratory disease	1.174 (0.622-1.174)	0.621		
Surgery	0.969 (0.530-1.770)	0.918		
Sepsis	0.820 (0.343-1.959)	0.655		
Trauma	0.558 (0.187-1.661)	0.294		
Other	2.490 (0.935-6.634)	0.068		
Comorbidities				
ACS	1.735 (0.753-3.996)	0.196		
AKI	0.873 (0.397-1.918)	0.735		
ALI	1.178 (0.481-2.883)	0.720		
ARDS	1.443 (0.659-3.158)	0.359		
Cirrhosis	0.701 (0.232-2.118)	0.529		
Diabetes mellitus	2.385 (1.159-4.908)	**0.018**	2.326 (1.055-5.130)	**0.036**
DIC	1.328 (1.36-12.998)	0.808		
Drinking	0.522 (0.242-1.125)	0.097		
Hypertension	0.832 (0.434-1.595)	0.580		
Malignant disease	1.116 (0.568-2.193)	0.749		
MOF	2.266 (1.146-4.482)	**0.019**	1.265 (0.590-2.715)	0.545
Smoking	0.656 (0.344-1.251)	0.201		
Stroke	1.067 (0.461-2.466)	0.880		
APACHE II	1.093 (1.052-1.135)	**<0.001**	1.065 (1.007-1.128)	**0.027**
Glucose	0.990 (0.928-1.056)	0.795		
Lactate	1.131 (1.065-1.200)	**<0.001**	1.093 (1.024-1.168)	**0.008**
qSOFA	1.547 (1.108-2.159)	**0.010**	0.855 (0.545-1.343)	0.497
SOFA	1.160 (1.083-1.242)	**<0.001**	1.057 (0.979-1.142)	0.157

**Table:** (a) Glucose < 7 mmol/L

Variable	Univariable regression		Multivariable regression	
	OR (95%-CI)	*p* value	OR (95%-CI)	*p* value
Age	0.507 (0.221-1.163)	0.109		
Gender	0.492 (0.214-1.132)	0.095		
Reason for ICU admission				
Cardiovascular disease	1.796 (0.701-4.601)	0.222		
Gastrointestinal disease	0.879 (0.364-2.121)	0.773		
Neurological disease	0.969 (0.300-3.315)	0.959		
Respiratory disease	1.447 (0.609-3.441)	0.403		
Surgery	1.360 (0.593-3.118)	0.467		
Sepsis	1.040 (0.320-3.383)	0.948		
Trauma	0.519 (0.112-2.406)	0.402		
Other	2.917 (0.877-9.695)	0.081		
Comorbidities				
ACS	0.914 (0.187-4.475)	0.911		
AKI	1.162 (0.423-3.192)	0.771		
ALI	0.874 (0.234-3.266)	0.841		
ARDS	2.619 (0.938-7.314)	0.066		
Cirrhosis	0.393 (0.048-3.199)	0.383		
Diabetes mellitus	3.363 (0.984-11.498)	0.053		
DIC	4.250 (0.258-70.045)	0.312		
Drinking	0.180 (0.041-0.798)	**0.024**	0.157 (0.018-1.399)	0.097
Hypertension	0.634 (0.238-1.690)	0.362		
Malignant disease	2.308 (0.882-6.043)	0.089		
MOF	2.550 (1.003-6.481)	**0.049**	1.443 (0.475-4.386)	0.517
Smoking	0.365 (0.139-0.962)	**0.042**	0.484 (0.115-2.041)	0.323
Stroke	0.613 (0.169-2.232)	0.458		
APACHE II	1.083 (1.027-1.142)	**0.003**	1.043 (0.969-1.123)	0.262
Lactate	1.126 (1.042-1.216)	**0.003**	1.093 (1.007-1.188)	**0.035**
qSOFA	1.356 (0.862-2.132)	0.188		
SOFA	1.147 (1.043-1.262)	**0.005**	1.104 (1.006-1.210)	**0.036**

**Table:** (b) 7 mmol/L ≤ glucose ≤ 9 mmol/L

Variable	Univariable regression		Multivariable regression	
	OR (95%-CI)	*p* value	OR (95%-CI)	*p* value
Age	0.507 (0.221-1.163)	0.109		
Gender	1.067 (0.301-3.785)	0.920		
Reason for ICU admission				
Cardiovascular disease	1.387 (0.310-6.216)	0.669		
Gastrointestinal disease	0.312 (0.087-1.115)	0.073		
Neurological disease	2.400 (0.489-11.772)	0.281		
Respiratory disease	0.927 (0.216-3.986)	0.919		
Surgery	0.732 (0.195-2.749)	0.644		
Sepsis	0.542 (0.059-4.956)	0.587		
Trauma	0.841 (0.155-4.554)	0.841		
Other	—	—		
Comorbidities				
ACS	1.629 (0.356-7.456)	0.530		
AKI	0.987 (0.179-5.450)	0.988		
ALI	4.200 (0.735-23.991)	0.107		
ARDS	0.841 (0.155-4.554)	0.841		
Cirrhosis	0.854 (0.087-8.383)	0.892		
Diabetes mellitus	2.891 (0.747-11.192)	0.124		
DIC	—	—		
Drinking	0.727 (0.136-3.880)	0.709		
Hypertension	0.738 (0.175-3.124)	0.680		
Malignant disease	0.137 (0.016-1.152)	0.067		
MOF	2.889 (0.672-12.415)	0.154		
Smoking	1.556 (0.395-6.131)	0.528		
Stroke	3.909 (0.494-30.942)	0.197		
APACHE II	1.144 (1.027-1.142)	**0.005**	1.126 (0.982-1.290)	0.089
Lactate	1.168 (0.928-1.470)	0.186		
qSOFA	1.953 (0.891-4.279)	0.095		
SOFA	1.229 (1.033-1.463)	**0.020**	1.041 (0.812-1.333)	0.753

**Table:** (c) Glucose > 9 mmol/L

Variable	Univariable regression		Multivariable regression	
	OR (95%-CI)	*p* value	OR (95%-CI)	*p* value
Age	1.019 (0.984-1.050)	0.288		
Gender	3.250 (0.965-10.950)	0.057		
Reason for ICU admission				
Cardiovascular disease	2.813 (0.718-11.021)	0.138		
Gastrointestinal disease	0.698 (0.214-2.278)	0.552		
Neurological disease	1.877 (0.509-6.921)	0.344		
Respiratory disease	0.977 (0.281-3.398)	0.971		
Surgery	0.642 (0.188-2.188)	0.479		
Sepsis	0.727 (0.145-3.636)	0.698		
Trauma	0	0.999		
Other	1.047 (0.109-10.014)	0.968		
Comorbidities				
ACS	3.333 (0.823-13.494)	0.091		
AKI	0.341 (0.041-2.840)	0.320		
ALI	0.571 (0.066-4.983)	0.613		
ARDS	0.492 (0.057-4.226)	0.518		
Cirrhosis	1.050 (0.202-5.460)	0.954		
Diabetes mellitus	1.513 (0.420-5.447)	0.526		
DIC	0	0.999		
Drinking	1.432 (0.439-4.673)	0.552		
Hypertension	1.391 (0.453-4.268)	0.564		
Malignant disease	1.324 (0.408-4.295)	0.641		
MOF	1.476 (0.353-6.166)	0.593		
Smoking	1.062 (0.332-3.404)	0.919		
Stroke	1.476 (0.353-6.166)	0.593		
APACHE II	1.087 (1.017-1.161)	**0.014**	1.056 (0.962-1.158)	0.252
Lactate	1.154 (1.035-1.285)	**0.010**	1.118 (0.997-1.255)	0.057
qSOFA	1.783 (0.940-3.383)	0.077		
SOFA	1.157 (1.026-1.305)	**0.017**	1.039 (0.876-1.233)	0.658

## Data Availability

The subsets of the data sets used and/or analyzed during the current study are available from the corresponding author on reasonable request.

## Abbreviations

ACS: Acute coronary syndrome
AKI: Acute kidney injury
ALI: Acute lung injury
APACHE II: Acute Physiology and Chronic Health Evaluation II
ARDS: Acute respiratory distress syndrome
AUROC: Area under receiver operating characteristic curve
CAD: Coronary artery disease
Cv-aCO_2_/Da-vO_2_: Ratio of venous-to-arterial carbon dioxide content difference to arterial-to-venous oxygen content difference
DE: Emergency department
DIC: Disseminated intravascular coagulation
ICU: Intensive care unit
MOF: Multiple organ failure
qSOFA: Quick Sequential Organ Failure Assessment
ROC: Receiver operating characteristic curve
SOFA: Sequential Organ Failure Assessment.

## Supplementary Materials

Supplementary Materials Table S1: patient number of 6 subgroups.
