# Supplementary material for: The Impact of Serum Glucose on the Predictive Value of Serum Lactate for Hospital Mortality in Critically Ill Surgical Patients
Source: Dis Markers. 2019 Nov 26;2019:1578502. doi: 10.1155/2019/1578502 (PMC6899272; doi:10.1155/2019/1578502)
Supplement: Supplementary Materials — Table S1: patient number of 6 subgroups. [file 1578502.f1.pdf]

Table S1 Patient number of 6 subgroups

	Lactate (mmol/L)	Glucose (mmol/L)	Number of patient
Subgroup 1	<1.45	<7	103
Subgroup 2	<1.45	>=7&<=9	41
Subgroup 3	<1.45	>9	55
Subgroup 4	>1.45	<7	45
Subgroup 5	>1.45	>=7&<=9	17
Subgroup 6	>1.45	>9	32
